# Supplementary material for: Physiological and pathophysiological control of synaptic GluN2B-NMDA receptors by the C-terminal domain of amyloid precursor protein
Source: eLife. 2017 Jul 6;6:e25659. doi: 10.7554/eLife.25659 (PMC5544428; doi:10.7554/eLife.25659)
Supplement: Supplementary file 1. — (A) AICD increases NMDAR, but not AMPAR, transmission in CA1 pyramidal neurons. (B) AICD regulates synaptic currents mediated by increasing synaptic GluN2B contribution. (C) Statistics (AICD regulates GluN2B mRNA levels). (D) APP knock down eliminates synaptic GluN2B NMDARs, an effect rescued by nuclear AICD delivery. (E) Physiological and pathological levels of AICD in the brain. (F) AICD perturbs synaptic signal integartion and discharge probability. (G) AICD perturbs synaptic signal integration and discharge probability by affecting the NMDAR-SK2 channel coupling. (H) LTP, but not LTD, is impaired in AICD neurons, a phenotype rescued by partial blockade of GluN2B subunits. DOI: http://dx.doi.org/10.7554/eLife.25659.013 [file elife-25659-supp1.pdf]

Supplementary file 1A - Statistics (AICD increases NMDAR, but not AMPAR, transmission in CA1 pyramidal neurons).														
**Experiment**	**cell designation**	**# animals**	**#cells**	**Measurement**	**Treatment**	**Average**	**s.e.m.**	**Units**	**Statistical Test**	**p value**	**F/t/z/R ETC value**	**pos hoc Test**	**p value**	**Figure**
**AMPAR/NMDAR ratio**	GFP	9	18	30 sweeps	_	3,99	0,59	_	One-way ANOVA	**p=0,0001**	F(6, 99)=5,068	Dunnet's (multiple comparisons)	GFP v AICD: <0,05<br>GFP v AICD-NLS: <0,01<br>GFP v APLP2-ICD: <0,05	2A
	AICD	13	16			2,35	0,26	_						
	AICD-NLS	14	22			1,81	0,19	_						
	AICDY682G	4	15			4,14	0,69	_						
	AICD-NES	3	13			4,94	0,93	_						
	APLP1-ICD	3	12			4,30	1,04	_						
	APLP2-ICD	4	10			1,52	0,20	_						
**mini AMPARs - Frequency**	GFP	5	11	10 min	_	0,50	0,12	Hz	One-way ANOVA	p=0,8958	F(2,25)=0,1105	_	_	2C
	AICD	4	11			0,44	0,08	Hz						
	AICD-NLS	4	6			0,49	0,09	Hz						
**mini AMPARs - Amplitude**	GFP	5	11	10 min	_	9,67	0,46	pA	One-way ANOVA	p=0,6354	F(2,25)=0,4619	_	_	2D
	AICD	4	11			10,41	0,74	pA						
	AICD-NLS	4	6			9,75	0,57	pA						
**Dual Patch AMPAR**	GFP	8	11	30 sweeps	_	107,00	18,44	pA	Wilcoxon matched-pairs two-tailed	p=0,956	_	_	_	2G
	AICD-NLS	6	9			147,10	15,75	pA						
**Dual Patch NMDAR**	GFP	6	8	30 sweeps	_	143,60	34,46	pA	Wilcoxon matched-pairs two-tailed	**p=0,008**	_	_	_	2I
	AICD-NLS	6	8			388,50	71,51	pA						

Supplementary file 1B - Statistics (AICD regulates synaptic currents mediated by increasing synaptic GluN2B contribution).

Experiment		cell designation	# animals	#cells	Measurement	Treatment	Average	s.e.m.	Units	Statistical Test	p value	F/t/z/R ETC value	pos hoc Test	p value	Figure
NMDAR kinetics	**τslow**	GFP	7	11	60 sweeps	CTR	226,25	33,05	ms	Two-way ANOVA	**Interaction p=0,0314 Treatment p=0,0213**	Interaction F(1,31)=5,080 Treatment F(1,31)=5,887	Tukey's (multiple comparisons)	GFP v AICD-NLS: <0,05 AICD-NLS v AICD-NLS Ifenprodil 5µM: <0,05	3A
		AICD-NLS	4	9		CTR	399,39	69,46	ms						
		GFP	5	8		Ifenprodil (5µM)	218,43	32,09	ms						
		AICD-NLS	3	7		Ifenprodil (5µM)	186,78	21,48	ms						
	**τfast**	GFP	7	11	60 sweeps	CTR	49,47	6,34	ms	Two-way ANOVA	Interaction p=0,5138 Treatment p=0,1552	Interaction F(1,31)=0,5138 Treatment F(1,31)=0,1552	_	_	3A
		AICD-NLS	4	9		CTR	53,11	4,17	ms						
		GFP	5	8		Ifenprodil (5µM)	45,01	5,61	ms						
		AICD-NLS	3	7		Ifenprodil (5µM)	41,25	4,45	ms						
	**τweighted**	GFP	7	11	60 sweeps	CTR	115,50	13,34	ms	Two-way ANOVA	**Interaction p=0,0035 Treatment p=0,0189**	Interaction F(1,31)=10,02 Treatment F(1,31)=6,140	Tukey's (multiple comparisons)	GFP v AICD-NLS: <0,05 AICD-NLS v AICD-NLS Ifenprodil 5µM: <0,01	3A
		AICD-NLS	4	9		CTR	192,77	25,32	ms						
		GFP	5	8		Ifenprodil (5µM)	127,65	17,08	ms						
		AICD-NLS	3	7		Ifenprodil (5µM)	93,05	11,85	ms						
**TAT-AICD-NLS**	**Vehicle**	_	5	8	last 10 min	Ifenprodil (5µM)	77,56	7,20	%	One-way-ANOVA	**p=0,0225**	F(4,33)=3,290	Dunnet's (multiple comparison)	vehicle v TAT-AICD-NLS (30nM): <0,05 vehicle v TAT-AICD-NLS (100nM): <0,05	3 D, E
	**10 nM**	_	4	6			69,12	11,29	%						
	**30nM**	_	4	8			47,19	7,43	%						
	**100nM**	_	4	9			45,93	5,47	%						
**Contribution of GluN2B to NMDAR-EPSCs at PND 32-40**	**in vivo AICD expression**	GFP	5	9	last 10 min	Ifenprodil (5µM)	18,70	8,32	%	One-way ANOVA	**p=0,0007**	F(7,58)=4,293	Uncorrected Fisher's LSD (multiple comparisons)	GFP v AICD-NLS: <0,05	3E
		AICD-NLS	3	5			48,15	6,35	%						
	**ex vivo AICD delivery**	vehicle	5	8			22,25	7,27	%						
		TAT-scAICD-NLS	4	8			20,05	9,07	%					TAT-scAICD-NLS v TAT-AICD-NLS: <0,01	
		TAT-AICD-NLS	7	14			58,66	4,14	%						
		TAT-AICD-NES	4	8			35,64	10,03	%					TAT-AICD-NLS v TAT-AICD-NES: <0,05	
		TAT-AICD-NLS + actinomycin	4	8			14,07	13,57	%					TAT-AICD-NLS v TAT-AICD-NLS + actisomycin: <0,001	
		TAT-AICD-NLS + anisomycin	4	8			21,13	9,28	%					TAT-AICD-NLS v TAT-AICD-NLS + anisomycin: <0,01	
**TAT-AICD nuclear localization**	**N2A APPKO cells**	TAT-AICD-NLS	3	55	APP fluorescence intensity (nucleus / cytoplasm)	_	0,66	0,02	ua	Unpaired t-test	**p<0,0001**	F(54,49)=1,964	_	_	3H
		TAT-AICD-NES	3	50			0,50	0,02	ua						

Supplementary file 1C - Statistics (AICD regulates GluN2B mRNA levels).

Experiment		cell designation	# animals	#cells	Measurement	Treatment	Average	s.e.m.	Units	Statistical Test	p value	F/t/z/R ETC value	pos hoc Test	p value	Figure
qPCR	APP C-Ter mRNA	synGFP	18	_	Fold change	_	1,00	0,02	ua	Unpaired t-test	**p<0,001**	t=8,461 df=35	_	_	4C
		synAICD	19	_			6,20	0,60	ua						
	GluN2B mRNA	synGFP	18	_			1,00	0,02	ua	Unpaired t-test	**p=0,006**	t=2,944 df=35	_	_	4D
		synAICD	19	_			1,10	0,02	ua						

Supplementary file 1D - Statistics (APP knock down eliminates synaptic GluN2B NMDARs, an effect rescued by nuclear AICD delivery).

Experiment		cell designation	# animals	#cells	Measurement	Treatment	Average	s.e.m.	Units	Statistical Test	p value	F/t/z/R ETC value	pos hoc Test	p value	Figure
APP expression	Hippocampal cell culture	shAPP inactive	_	36	Mean APP fluorescence intensity	GFP-	86,52	4,73	ua	Two-way ANOVA	Interaction p=0,046 shAPP p<0,0001	Interaction F(1,125)=8,312 shAPP F(1,125)=57,94	Tukey's (multiple comparisons)	shAPP inactive GFP+ vs shAPP active GFP+ <0,0001	**5B**
			_	42		GFP+	64,14	4,20	ua						
		shAPP active	_	25		GFP-	78,51	5,84	ua					shAPP active GFP- vs shAPP active GFP+ <0,0001	
			_	26		GFP+	28,84	3,31	ua						
	hHippocampal slices	shAPP inactive	3	15		_	47,67	3,96	ua	Man-whitney test	p<0,0001	_	_	_	**5D**
		shAPP inactive	3	28		_	8,02	0,88	ua						
NMDAR kinetics	τfast	shAPP-inactive	6	9	60 sweeps	CTR	38,63	4,81	ms	Two-way ANOVA	Interaction p=0,072 Treatment p=0,6486	Interaction F(1,34)=3,495 Treatment F(1,34)=0,2113	_	_	5F
		shAPP-active	8	14		CTR	30,19	2,62	ms						
		shAPP-inactive	6	8		Ifenprodil (5µM)	30,14	3,57	ms						
		shAPP-active	7	7		Ifenprodil (5µM)	35,33	3,01	ms						
	τslow	shAPP-inactive	6	9	60 sweeps	CTR	186,12	14,94	ms	Two-way ANOVA	**Interaction p=0,002 Treatment p=0,0019**	Interaction F(1,34)=11,16 Treatment F(1,34)=11,37	Tukey's (multiple comparisons)	shAPP-inactive v shAPP-active < 0,01	
		shAPP-active	8	14		CTR	133,20	7,45	ms					shAPP-inactive v shAPP-inactive Ifen < 0,001	
		shAPP-inactive	6	8		Ifenprodil (5µM)	110,29	10,55	ms						
		shAPP-active	7	7		Ifenprodil (5µM)	132,85	12,10	ms						
	τweighted	shAPP-inactive	6	9	60 sweeps	CTR	102,28	11,53	ms	Two-way ANOVA	**Interaction p=0,011 Treatment p=0,004**	Interaction F(1,34)=7,274 Treatment F(1,34)=9,333	Tukey's (multiple comparisons)	shAPP-inactive v shAPP-active < 0,01	
		shAPP-active	8	14		CTR	71,21	2,73	ms					shAPP-inactive v shAPP-inactive Ifen < 0,01	
		shAPP-inactive	6	8		Ifenprodil (5µM)	68,79	5,76	ms						
		shAPP-active	7	7		Ifenprodil (5µM)	63,30	5,10	ms						
Contribution of GluN2B to NMDAR-EPSCs at PND 7-9	**ACSF**	shAPP-inactive	6	8	last 10 min	Ifenprodil (5µM)	53,89	6,81	%	One-way ANOVA	**p=0,0001**	F(3,26)=10,27	Tukey's (multiple comparisons)	shAPP inactive v shAPP active <0,001	5H
	**ACSF**	shAPP-active	8	7			-4,04	13,46	%					shAPP-active v shAPP active TAT-AICD-NLS <0,01	
	**TAT-AICD-NLS**	shAPP-active	8	8			48,57	6,26	%						
	**TAT-scAICD-NLS**	shAPP-active	8	6			8,38	9,40	%					shAPP-active TAT-AICD-NLS v shAPP-active TATscAICD-NLS <0,05	
	**TAT-AICD-NLS**	shAPP-inactive	3	5			37,48	7,91	%						

Supplementary file 1E - Statistics (Physiological and pathological levels of AICD in the brain).

Experiment	cell designation	# animals	#cells	Measurement	Treatment	Average	s.e.m.	Units	Statistical Test	p value	F/t/z/R ETC value	pos hoc Test	p value	Figure
AICD quantification	TAT-AICD-NLS	2	_	AICD (pg/µg)	_	10,28	0,40	pg/µg	One-way ANOVA	**p=0,0002**	F(2,17)=14,40	Tukey's (multiple comparisons)	WT v Tg2576 <0,001	6C
	WT	8	_			4,65	0,78	pg/µg						
	Tg2576	8	_			24,67	4,11	pg/µg						

Supplementary file 1F - Statistics (AICD perturbs synaptic signal integartion and discharge probability).

Experiment		cell designation	# animals	#cells	Measurement	Treatment	Average	s.e.m.	Units	Statistical Test	p value	F/t/z/R ETC value	pos hoc Test	p value	Figure
**Stimulus Frequency 0.1Hz**	**1st Pulse**	GFP	4	10	EPSP amplitude	_	3,69	0,49	mV	Multiple t-Test GFP vs AICD-NLS	p=0,5348	t(16)=0,6344	_	_	7A
		AICD-NLS	4	8			3,21	0,58	mV						
	**2nd Pulse**	GFP	4	10			2,87	3,33	mV		p=0,4744	t(16)=0,7326	_	_	
		AICD-NLS	4	8			3,33	0,52	mV						
	**3rd Pulse**	GFP	4	10			3,44	0,51	mV		p=0,6021	t(16)=0,5319	_	_	
		AICD-NLS	4	8			3,07	0,46	mV						
	**4th Pulse**	GFP	4	10			3,66	0,65	mV		p=0,7738	t(16)=0,2923	_	_	
		AICD-NLS	4	8			3,42	0,44	mV						
	**5th Pulse**	GFP	4	10			3,91	0,64	mV		p=0,8834	t(16)=0,1488	_	_	
		AICD-NLS	4	8			4,03	0,45	mV						
**Stimulus Frequency 1Hz**	**1st Pulse**	GFP	4	10	EPSP amplitude	_	3,12	0,59	mV	Multiple t-Test GFP vs AICD-NLS	p=0,5706	t(17)=0,5783	_	_	7B
		AICD-NLS	4	9			3,65	0,68	mV						
	**2nd Pulse**	GFP	4	10			3,24	0,62	mV		p=0,9991	t(17)=0,0011	_	_	
		AICD-NLS	4	9			3,24	0,55	mV						
	**3rd Pulse**	GFP	4	10			3,80	0,76	mV		p=0,5347	t(17)=0,6337	_	_	
		AICD-NLS	4	9			4,52	0,83	mV						
	**4th Pulse**	GFP	4	10			3,17	0,61	mV		p=0,6029	t(17)=0,5301	_	_	
		AICD-NLS	4	9			3,63	0,62	mV						
	**5th Pulse**	GFP	4	10			3,96	0,61	mV		p=9670	t(17)=0,0420	_	_	
		AICD-NLS	4	9			3,92	0,65	mV						
**Stimulus Frequency 10Hz**	**1st Pulse**	GFP	7	22	EPSP amplitude	_	5,61	0,42	mV	Two-Way ANOVA	**p = 0,0153**	F(2, 57) = 4,506	Dunnet's (multiple comparision)		7C
		AICD	4	10			5,31	0,39	mV					GFP v AICD-NLS >0,05	
		AICD-NLS	8	28			4,57	0,94	mV					GFP v AICD >0,05	
	**2nd Pulse**	GFP	7	22			9,41	0,89	mV						
		AICD	4	10			7,12	0,73	mV					GFP v AICD-NLS <0,05	
		AICD-NLS	8	28			6,85	1,62	mV					GFP v AICD >0,05	
	**3rd Pulse**	GFP	7	22			8,91	0,90	mV						
		AICD	4	10			5,96	0,71	mV					GFP v AICD-NLS <0,01	
		AICD-NLS	8	28			5,77	1,47	mV					GFP v AICD <0,05	
	**4th Pulse**	GFP	7	22			8,64	0,89	mV						
		AICD	4	10			4,95	0,62	mV					GFP v AICD-NLS <0,01	
		AICD-NLS	8	28			5,39	1,35	mV					GFP v AICD <0,01	
	**5th Pulse**	GFP	7	22			8,24	0,85	mV						
		AICD	4	10			4,66	0,65	mV					GFP v AICD-NLS <0,01	
		AICD-NLS	8	28			4,69	0,96	mV					GFP v AICD <0,01	
**Discharge Probablility**	**1st Pulse**	GFP	4	12	Action Potential Occurence =1 Action Potential failure = 0	_	0,00	0,00	-	Multiple t-Test GFP vs AICD-NLS	**p = 0,00027**	F(2, 32) = 7,149	Dunnet's (multiple comparision)		7D
		AICD	4	10			0,00	0,00						GFP v AICD-NLS >0,05	
		AICD-NLS	6	16			0,00	0,00	-					GFP v AICD >0,05	
	**2nd Pulse**	GFP	4	12			0,67	0,17	-						
		AICD	4	10			0,44	0,18						GFP v AICD-NLS >0,05	
		AICD-NLS	6	16			0,47	0,13	-					GFP v AICD >0,05	
	**3rd Pulse**	GFP	4	12			0,89	0,11	-						
		AICD	4	10			0,89	0,11						GFP v AICD-NLS >0,05	
		AICD-NLS	6	16			0,53	0,13	-					GFP v AICD >0,05	
	**4th Pulse**	GFP	4	12			1,00	0,00	-						
		AICD	4	10			0,67	0,17						GFP v AICD-NLS >0,05	
		AICD-NLS	6	16			0,65	0,12	-					GFP v AICD >0,05	
	**5th Pulse**	GFP	4	12			1,00	0,00	-						
		AICD	4	10			0,22	0,15						GFP v AICD-NLS <0,01	
		AICD-NLS	6	16			0,47	0,13	-					GFP v AICD <0,01	

Supplementary file 1G - Statistics (AICD perturbs synaptic signal integration and discharge probability by affecting the NMDAR-SK2 channel coupling).

Experiment		cell designation	# animals	#cells	Measurement	Treatment	Average	s.e.m.	Units	Statistical Test	p value	F/t/z/R ETC value	pos hoc Test	p value	Figure
**Effect of Apamin on EPSPs**		GFP	3	8	last 10 minutes	placebo	178,48	18,77	%	Two-way ANOVA	**Treatment p=0,0025 Virus p=0,0034**	Treatment F(1,25)=11,25 Virus F(1,25)=10,44	Tukey's (multiple comparisons)	GFP v AICD-NLS: <0,05<br>AICD-NLS v AICD-NLS Ifenprodil: <0,05	8C
		AICD-NLS	3	8			294,92	47,73	%						
		GFP	4	4	last 10 minutes	Ifenprodil (5µM)	183,71	18,24	%						
		AICD-NLS	3	8			181,45	12,59	%						
**Stimulus Frequency 10Hz**	**1st Pulse**	GFP	7	22	EPSP amplitude	Placebo	5,61	0,42	mV	Two-Way ANOVA	Ifenprodil rescue: **p=0,0432** apamin rescue: **p=0,0344**	Ifenprodil rescue: F(4, 216) = 18,93 apamin rescue: F(8, 216) = 23,40	–	–	8D 8F
		AICD-NLS	8	28		Placebo	4,57	0,94	mV						
		AICD-NLS	3	7		Ifenprodil (300nM)	5,23	0,43	mV						
		AICD-NLS	3	7		Apamin (100nM)	5,40	0,79	mV						
	**2nd Pulse**	GFP	7	22		Placebo	9,41	0,89	mV				–	–	
		AICD-NLS	8	28		Placebo	6,85	1,62	mV						
		AICD-NLS	3	7		Ifenprodil (300nM)	7,76	0,88	mV						
		AICD-NLS	3	7		Apamin (100nM)	9,84	1,38	mV						
	**3rd Pulse**	GFP	7	22		Placebo	8,91	0,90	mV				–	–	
		AICD-NLS	8	28		Placebo	5,77	1,47	mV						
		AICD-NLS	3	7		Ifenprodil (300nM)	8,69	0,92	mV						
		AICD-NLS	3	7		Apamin (100nM)	8,67	1,69	mV						
	**4th Pulse**	GFP	7	22		Placebo	8,64	0,89	mV				–	–	
		AICD	8	28		Placebo	5,39	1,35	mV						
		AICD-NLS	3	7		Ifenprodil (300nM)	7,79	1,04	mV						
		AICD-NLS	3	7		Apamin (100nM)	8,11	1,82	mV						
	**5th Pulse**	GFP	7	22		Placebo	8,24	0,85	mV				Dunnet's (multiple comparision)	GFP v AICD-NLS <0,01<br>GFP v AICD-NLS ifenprodil >0,05<br>GFP v AICD-NLS apamin >0,05	
		AICD-NLS	8	28		Placebo	4,69	0,96	mV						
		AICD-NLS	3	7		Ifenprodil (300nM)	7,51	0,99	mV						
		AICD-NLS	3	7		Apamin (100nM)	8,52	1,74	mV						
**Discharge Probablility**	**1st Pulse**	GFP	4	9	Action Potential Occurence =1 Action Potential failure = 0	Placebo	0,00	0,00	-	Two-Way ANOVA	Ifenprodil rescue: **p=0,0135** apamin rescue: **p=0,0447**	Ifenprodil rescue: F(4, 124) = 24,73 apamin rescue: F(4, 116) = 20,79	–	–	8E 8G
		AICD-NLS	4	17		Placebo	0,00	0,00	-						
		AICD-NLS	3	8		Ifenprodil (300nM)	0,00	0,00	-						
		AICD-NLS	3	7		Apamin (100nM)	0,00	0,00	-						
	**2nd Pulse**	GFP	4	9		Placebo	0,67	0,17	-				–	–	
		AICD-NLS	4	17		Placebo	0,47	0,13	-						
		AICD-NLS	3	8		Ifenprodil (300nM)	0,38	0,18	-						
		AICD-NLS	3	7		Apamin (100nM)	0,67	0,21	-						
	**3rd Pulse**	GFP	4	9		Placebo	0,89	0,11	-				–	–	
		AICD	4	17		Placebo	0,53	0,13	-						
		AICD-NLS	3	8		Ifenprodil (300nM)	0,88	0,13	-						
		AICD-NLS	3	7		Apamin (100nM)	0,83	0,17	-						
	**4th Pulse**	GFP	4	9		Placebo	1,00	0,00	-				–	–	
		AICD	4	17		Placebo	0,65	0,12	-						
		AICD-NLS	3	8		Ifenprodil (300nM)	0,88	0,13	-						
		AICD-NLS	3	7		Apamin (100nM)	0,83	0,17	-						
	**5th Pulse**	GFP	4	9		Placebo	1,00	0,00	-				Dunnet's (multiple comparision)	GFP v AICD-NLS <0,001<br>GFP v AICD-NLS ifenprodil >0,05<br>GFP v AICD-NLS apamin >0,05	
		AICD-NLS	4	17		Placebo	0,35	0,13	-						
		AICD-NLS	3	8		Ifenprodil (300nM)	0,88	0,13	-						
		AICD-NLS	3	7		Apamin (100nM)	0,83	0,17	-						

Supplementary file 1H - Statistics (LTP, but not LTD, is impaired in AICD neurons, a phenotype rescued by partial blockade of GluN2B subunits).

Experiment	cell designation	# animals	#cells	Measurement	Treatment	Average	s.e.m.	Units	Statistical Test	p value	F/t/z/R ETC value	pos hoc Test	p value	Figure
**NMDAR dependent LTD**	GFP	5	7	last 10 minutes	_	56,87	8,75	%	One-way ANOVA	p=0,9538	F(2,14)=0,047	_	_	9B
	AICD	3	4			57,09	16,22	%						
	AICD-NLS	4	7			60,47	4,94	%						
**NMDAR dependent LTP**	GFP	7	10	last 10 minutes	_	191,00	29,58	%	One-way ANOVA	**p=0,0049**	F(2,22)=6,838	Tukey's (multiple comparisons)	GFP v AICD: <0,05<br>GFP v AICD-NLS: <0,01	9D
	AICD	3	6			94,52	9,55	%						
	AICD-NLS	7	9			98,08	7,86	%						
**LTD induction**	GFP	5	7	Sn/S1 (last 10)	_	107,4	8,73	%	Student t-test	p=0,2194	t =1,367	_	_	9-S1B
	AICD-NLS	4	7			133,10	16,62	%						
**LTP induction**	GFP	7	10	Sn/S1 (last 10)	placebo	100,2	15,14	%	Two-way ANOVA	**Interaction p=0,0030**	Interaction F(1,27)=10,65	Tukey's (multiple comparisons)	GFP placebo v AICD-NLS placebo: <0,01<br>AICD-NLS placebo v AICD-NLS Ifenprodil 300nM: <0,05	9-S1D
	AICD-NLS	7	9			44,20	6,18	%						
	GFP	3	6	Sn/S1 (last 10)	Ifenprodil 300nM	75,64	7,66	%						
	AICD-NLS	4	5			89,37	6,18	%						
**Effect of NR2B antagonist on NMDAR dependent LTP**	GFP	7	10	last 10 minutes	placebo	190,99	29,58	%	Two-way ANOVA	**Interaction p=0,0087**<br>**Treatment p=0,0109**	Interaction F(4,44)=3,882<br>Treatment F(4,44)=3,711	Tukey's (multiple comparisons)	GFP v AICD-NLS: <0,05<br>AICD-NLS vs AICD-NLS Ifenprodil 300nM: <0,05	9F
	AICD-NLS	7	9			98,08	7,86	%						
	GFP	3	5	last 10 minutes	Ifenprodil 30nM	185,88	22,19	%						
	AICD-NLS	3	5			107,92	12,70	%						
	GFP	3	6	last 10 minutes	Ifenprodil 300nM	132,36	21,31	%						
	AICD-NLS	4	5			209,90	28,62	%						
	GFP	3	4	last 10 minutes	Ifenprodil 1μM	126,37	9,85	%						
	AICD-NLS	3	3			140,07	18,02	%						
	GFP	2	3	last 10 minutes	Ifenprodil 5μM	68,18	18,70	%						
	AICD-NLS	2	3			86,06	11,88	%						
